# NIM811, a nonimmunosuppressive cyclosporine analogue, suppresses collagen production and enhances collagenase activity in hepatic stellate cells

**DOI:** 10.1111/j.1478-3231.2007.01560.x

**Published:** 2007-11

**Authors:** Motoyuki Kohjima, Munechika Enjoji, Nobito Higuchi, Kazuhiro Kotoh, Masaki Kato, Ryoiichi Takayanagi, Makoto Nakamuta

**Affiliations:** 1 Department of Medicine and Bioregulatory Science, Graduate School of Medical Sciences, Kyushu University Fukuoka, Japan; 2 Department of Gastroenterology, National Hospital Organization Kyushu Medical Center Fukuoka, Japan

**Keywords:** cyclosporine analogue, hepatic fibrosis, hepatic stellate cell, NIM811, TGF-β

## Abstract

**Background/Aims:**

A recent decrease in patient survival has been reported among hepatitis C virus (HCV)-infected liver transplant recipients and this may be attributable to progression of fibrosis. We reported previously that cyclosporine suppressed the proliferation of, and collagen production in, hepatic stellate cells (HSCs). Here, we investigated the effects of NIM811, a cyclosporine analogue, on cell growth, collagen production and collagenase activity in HSCs.

**Methods:**

Rat HSCs and human HSC-derived TWNT-4 cells were cultured for the study. The expression of collagen, matrix metalloproteinase 1 (MMP-1) and tissue inhibitor of metalloproteinase-1 (TIMP-1) and collagenase activity was evaluated. Cell proliferation and apoptosis were measured. Phosphorylation of mitogen-activated protein kinases (MAPKs), Smad2 and Smad3 was evaluated. The expression of the tumour growth factor-β (TGF-β)-receptor and Smad7 genes was also evaluated.

**Results:**

NIM811, as well as cyclosporine, suppressed the transcription and synthesis of collagen and stimulated the production of MMP-1 with a concomitant enhancement of collagenase activity, although it did not change the expression of TIMP-1. NIM811 inhibited proliferation without induction of apoptosis. In the MAPKs and TGF-β signalling pathways, NIM811 enhanced the phosphorylation of JNK and p38, but not extracellular signal-regulated kinases 1 and 2, and suppressed the phosphorylation of Smad2 and Smad3, accompanied by increased Smad7 transcription and decreased TGF-β-receptor transcription.

**Conclusion:**

These findings demonstrate that NIM811 not only suppresses collagen production and proliferation but also increases collagenase activity. These effects are accompanied by inhibition of TGF-β signalling pathways.

In liver transplantation, a significant decline in patient survival, caused by rapid progression to decompensated graft cirrhosis, is an important clinical issue for hepatitis C virus (HCV)-positive patients, whereas there has been an improvement in the survival of HCV-negative patients (1–5). Progression to cirrhosis has been estimated to occur in up to 30% of patients at 5 years after transplantation, and the rate of disease progression has been greater in patients who underwent transplantation in recent years compared with those who underwent transplantation a longer period of time ago (1, 2, 4). Several variables, including donor age, degree of immunosuppression and viral load, are implicated in the recent increase in progression (2, 3). Indeed, Berenguer *et al.* (3, 5) reported that HCV-infected patients who received immunosuppressive therapy with cyclosporine were less likely to develop graft cirrhosis than those who received tacrolimus, indicating that the choice of calcineurin inhibitor may influence disease progression (6).

Hepatic stellate cells (HSCs) are nonparenchymal liver cells that have a characteristic stellate morphology and reside in the perisinusoidal space of Disse (7). Following liver injury, HSCs undergo transdifferentiation to an activated myofibroblastic phenotype and express α-smooth muscle actin. Activated HSCs then proliferate and produce extracellular matrices (ECM) such as collagens (7, 8). Previously, we evaluated the effects of immunosuppressive drugs, such as cyclosporine and tacrolinus, on HSCs. Cell proliferation and collagen production were suppressed by cyclosporine but not by tacrolimus, indicating that cyclosporine potentially has an anti-fibrogenic effect (9).

Cyclosporine is an immunosuppressive cyclic undecapeptide, and binds with nanomolar affinity to cyclophilins. The complex of cyclosporine and cyclophilin A inhibits calcineurin, a calcium-dependent phosphatase that regulates the expression of various cytokine genes in activated T-lymphocytes (10–12). NIM811 is a four-substituted cyclosporine that does not bind to cyclophilin A and therefore lacks immunosuppressive activity; however, it retains the ability to bind other cyclophilins, such as cyclophilin B (13). Recently, NIM811, as well as cyclosporine, showed a suppressive effect on HCV at the RNA and protein levels in an HCV subgenomic replicon cell culture system (14). In this study, we investigated the effects of NIM811 on proliferation, collagen production and collagenase activity in HSCs *in vitro*. We demonstrated that NIM811 not only suppressed proliferation and collagen production but also enhanced collagenase activity in HSCs, indicating that NIM811 is a potential candidate for anti-fibrosis therapy.

## Materials and methods

### Cell culture

Hepatic stellate cells were isolated from the liver of male Wistar rats by sequential *in situ* perfusion with collagenase and digestion with pronase, followed by centrifugation in a double-layered (17%/11.5%) metrizamide solution (Sigma Chemical, St Louis, MO, USA), as described previously (15). HSCs were cultured in Dulbecco's modified Eagle's medium (DMEM) with 10% foetal calf serum (FCS). The experiments described in this study were performed on cells between the third and fourth serial passages. Because commercial kits for the measurement of mouse or rat matrix metalloproteinase 1 (MMP-1) and tissue inhibitor of metalloproteinase-1 (TIMP-1) were not available, we used TWNT-4 cells, a human cell line derived from HSCs (16), to evaluate the effects of NIM811 on MMP-1 and TIMP-1. TWNT-4 cells were cultured in DMEM with 10% FCS as reported previously (16). NIM811 was donated by Novartis Pharma AG (Basel, Switzerland). NIM811 was dissolved in DMEM and added to the cultures. The cell viability of HSCs was more than 90% under serum-free conditions for 24 h in the presence of 2 mM NIM811 under serum-free conditions.

### Type I collagen assay

Cultured HSCs were incubated in a serum-free medium in the presence or absence of NIM811 for 24 h. Type I collagen was determined in culture media by ELISA as described previously (17). Anti-rat type I collagen antibody (LSL, Tokyo, Japan) was used as the primary antibody and peroxidase-conjugated goat-anti-rabbit IgG (Organon Teknika Corporation, Durham, NC, USA) was used as the secondary antibody. Rat tail tendon collagen type I (Advance Biofactures Corporation, Lymbrook, NY, USA) was used as the standard.

### Matrix metalloproteinase 1, tissue inhibitor of metalloproteinase-1 and collagenase assay

Cultured TWNT-4 cells were incubated in a serum-free medium in the presence or absence of NIM811 for 24 h. MMP-1 and TIMP-1 productions were determined in culture media by ELISA using a Biotrak ELISA system for human MMP-1 (Amersham Biosciences, Piscataway, NJ, USA) and an hTIMP-1 kit (Daiichi Fine Chemical Co. Ltd, Toyama, Japan) respectively (18). Active MMP-1 and pro-MMP-1 in culture media were determined using an MMP-1 Biotrak Activity Assay System (Amersham) (18).

### Analysis of gene expression using real-time reverse transcriptase-polymerase chain recation

Total RNA was prepared with Trizol reagent (Invitrogen, Carlsbad, CA, USA) from TWNT-4 cells that were maintained in either the presence or absence of NIM811 in 10% FCS for 24 h. cDNA was synthesized from 1.0 μg RNA with GeneAmp^™^ RNA PCR (Applied Biosystems, Branchburg, NJ, USA) using random hexamers. Real-time PCR was performed using LightCycler-FastStart DNA Master SYBR Green 1 (Roche, Tokyo, Japan) as described previously (19). The reaction mixture (20 μL) contained LightCycler-FastStart DNA Master SYBR Green 1, 4 mM MgCl_2_, 0.5 μM of the upstream and downstream PCR primers and 2 μL of the first-strand cDNA as a template. To control for variations in the reactions, all PCRs were normalized against glyceraldehyde-3-phosphate dehydrogenase (GAPDH) expression. The primers used were as follows: 5′-AGGGTGAGACAGGCGAACAG-3′ (forward primer) and 5′-CTCTTGAGGTGGCTGGGGCA-3′ (reverse primer) for human type I collagen α1 chain (GenBank^™^ accession number NM000088) (20); 5′-GATCATCGGGACAACTCTCCT-3′ (forward primer) and 5′-TCCGGGTAGAAGGGATTTGTG-3′ (reverse primer) for MMP-1 (GenBank^™^ accession number NM002421) (21); 5′-TTCTGCAATTCCGACCTCGT-3′ (forward primer) and 5′-TCCGTCCACAAGCAATGAGT-3′ (reverse primer) for TIMP-1 (Ref. 3; GenBank^™^ accession number NM003254) (22); 5′-GGATCTCAGGCATTCCTCGG-3′ (forward primer) and 5′-CAGTATGCCACCACGCACCA-3′ (reverse primer) for Smad7 (23); and 5′-GGCCGTTTGTATGTGCACCCTC-3′ (forward primer) and 5′-GGGCGATCTAATGAAGGGTCC-3′ (reverse primer) for TGF-β-receptor I (TGF-β-RI) (24).

### Analysis of bromodeoxyuridine incorporation

Hepatic stellate cell incorporation of bromodeoxyuridine (BrdU) was measured using a cell proliferation ELISA (Roche Diagnostics GmbH, Mannheim, Germany) as described previously (25). Briefly, subconfluent HSCs were serum starved for 24 h. They were then washed with DMEM and incubated for 24 h with BrdU in DMEM with 10% FCS in the presence or absence of NIM811. After labelling the cells with BrdU, cellular DNA was digested and incubated with the anti-BrdU antibody conjugated with peroxidase. BrdU incorporation was estimated by measuring the fluorescence intensity of the supernatant at 450 nm (excitation) and 690 nm (emission).

### Analysis of apoptosis

Hepatic stellate cells were maintained in either the presence or absence of NIM811 under serum-free conditions for 24 h. Cells were fixed for 30 min in 4% paraformaldehyde/PBS at room temperature, and permeabilized for 5 min in PBS containing 0.2% Triton X-100 at 4°C. Cells were then stained with Hoechst 33 342 and analysed by the terminal deoxynucleotidyl Transferase Biotin-dUTP nick end labeling method using an *in situ* Cell Death Detection Kit (Roche) according to the manufacturer's instructions. The samples were visualized with an LSM 510 confocal laser scanning microscope (Carl Zeiss, Jena, Germany). At least 100 cells from three independent experiments and from three different cell preparations were counted for each condition.

### Western blot analysis for phospho- and nonphospho-mitogen-activated protein kinases

Western blot analysis was basically performed essentially as described previously (26). After starving HSCs for 24 h, they were treated with or without NIM811 for 2 h or mock treated. Whole-cell lysates containing 1 × 10^7^ TWNT-4 cells were prepared in 100 mL sodium dodecyl sulphate-polyacrylamide gel electrophoresis sample buffer. Protein lysates were subjected to 12% SDS-PAGE, transferred to a polyvinylidene difluoride membrane (Millipore, Bedford, MA, USA) and probed with the primary antibodies for extracellular signal-regulated kinases 1 and 2 (ERK1/2) mitogen-activated protein kinase (MAPK), phospho-ERK1/2 MAPK (Thr202/Tyr204), Jun N-terminal kinase (JNK), phospho-JNK (Thr183/Tyr185), p38 MAPK or phospho-p38 MAPK (Thr180/Tyr182) (New England Biolabs, Beverly, MA, USA). Antibody binding was detected using peroxidase-linked anti-rabbit IgG (Amersham Pharmacia Biotech, Piscataway, NJ, USA) as the secondary antibody. The blots were developed using ECL-plus (Amersham Pharmacia Biotech) to visualize the antibodies. The levels of ERK1/2 MAPK, phosphorylated-ERK1/2 MAPK, JNK, phosphorylated-JNK, p38 MAPK and phosphorylated-p38 MAPK were quantified by densitometry using an optical scanner system. For comparison, the ratios of phosphorylated ERK1/2, JNK and p38 MAPK to nonphosphorylated ERK1/2, JNK and p38 MAPK, respectively, were calculated from the densitometric data.

### Western blot analysis for phospho- and nonphospho-Smad2 and Smad3

Western blot analysis was performed as described above, probed with the primary antibody for Smad2, phospho-Smad2 (Thr/Tyr), Smad3 or phospho-Smad3 (Thr/Tyr) (Cell Signaling Technology, Danvers, MA, USA). For comparison, the ratios of phosphorylated Smad2 and Smad3 to nonphosphorylated Smad2 and Smad3, respectively, were calculated from the densitometric data.

### Statistical analysis

All results are shown as the mean±SEM. Comparisons were made using one-way anova, followed by Scheffe's test or the Mann–Whitney test.

## Results

### Effects of NIM811 on type I collagen accumulation, matrix metalloproteinase 1 and tissue inhibitor of metalloproteinase-1 production and collagenase activity

**Fig. 1:**
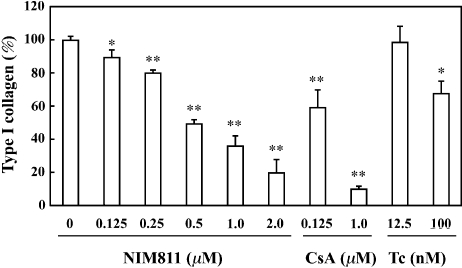
Effects of NIM811, cyclosporine (CsA) and tacrolimus (Tc) on the accumulation of type I collagen in rat hepatic stellate cells (HSCs). NIM811, as well as cyclosporine, caused a concentration-dependent inhibition of type I collagen production. In contrast, tacrolimus did not show these suppressive effects at a clinically relevant concentration (i.e. 12.5 nM, equivalent to 10 ng/mL). The data represent mean±SEM from five independent experiments. ^*^and ^**^Statistically significant differences (*P*<0.05 and <0.01 respectively) compared with HSCs in the absence of NIM811, CsA and Tc.

To assess the effect of NIM811 on ECM production by HSCs, we determined type I collagen concentrations in culture media after adjusting the number of rat HSCs. Treatment of the cells with increasing concentrations of NIM811, as well as cyclosporine, led to a concentration-dependent suppression of collagen accumulation; 0.5 mM NIM811 reduced collagen accumulation by approximately 50% (Fig. 1). As reported previously (9), cyclopsorine at the clinically relevant concentration of 0.125 mM (150 ng/mL) reduced collagen production by approximately 50%, whereas tacrolimus at the clinically relevant concentration of 12.5 nM (10 ng/mL) did not reduce collagen production significantly (Fig. 1).

**Fig. 2:**
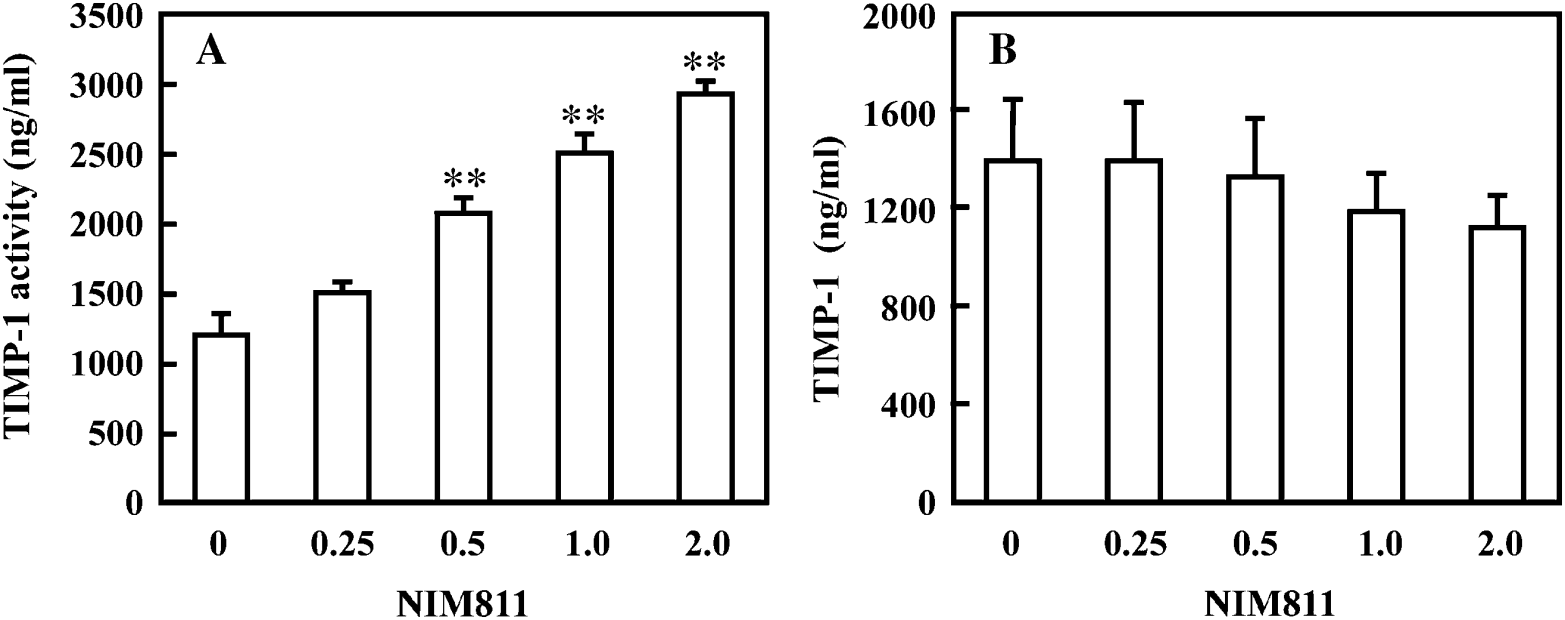
Effects of NIM811 on matrix metalloproteinase 1 (MMP-1) activity (collagenase activity) and tissue inhibitor of metalloproteinase-1 (TIMP-1) production in TWNT-4 cells. NIM811 caused a concentration-dependent enhancement of MMP-1 activity (collagenase activity) (A). NIM811 also tended to reduce TIMP-1 production in a concentration-dependent manner (B). The data represent mean±SEM from five independent experiments. ^*^and ^**^Statistically significant differences (*P*<0.05 and <0.01 respectively) compared with hepatic stellate cells (HSCs) in the absence of NIM811.

Because collagenase activity affects accumulation of type I collagen, we evaluated the effects of NIM811 on collagenase activity in TWNT-4 cells. NIM811 led to a concentration-dependent increase in collagenase activity (active MMP-1); in the presence of 0.5 mM NIM811, collagenase activity increased roughly two-fold (Fig. 2A). Because collagenase activity is regulated by the balance between MMP-1 and TIMP-1, we also evaluated TIMP-1 production in TWNT-4 cells. NIM811 tended to reduce TIMP-1 production in a concentration-dependent manner; however, NIM811 did not reduce TIMP-1 production significantly, even at a concentration of 2.0 mM (Fig. 2B).

### Effects of NIM811 on gene expression of type I collagen, matrix metalloproteinase 1 and tissue inhibitor of metalloproteinase-1

**Fig. 3:**
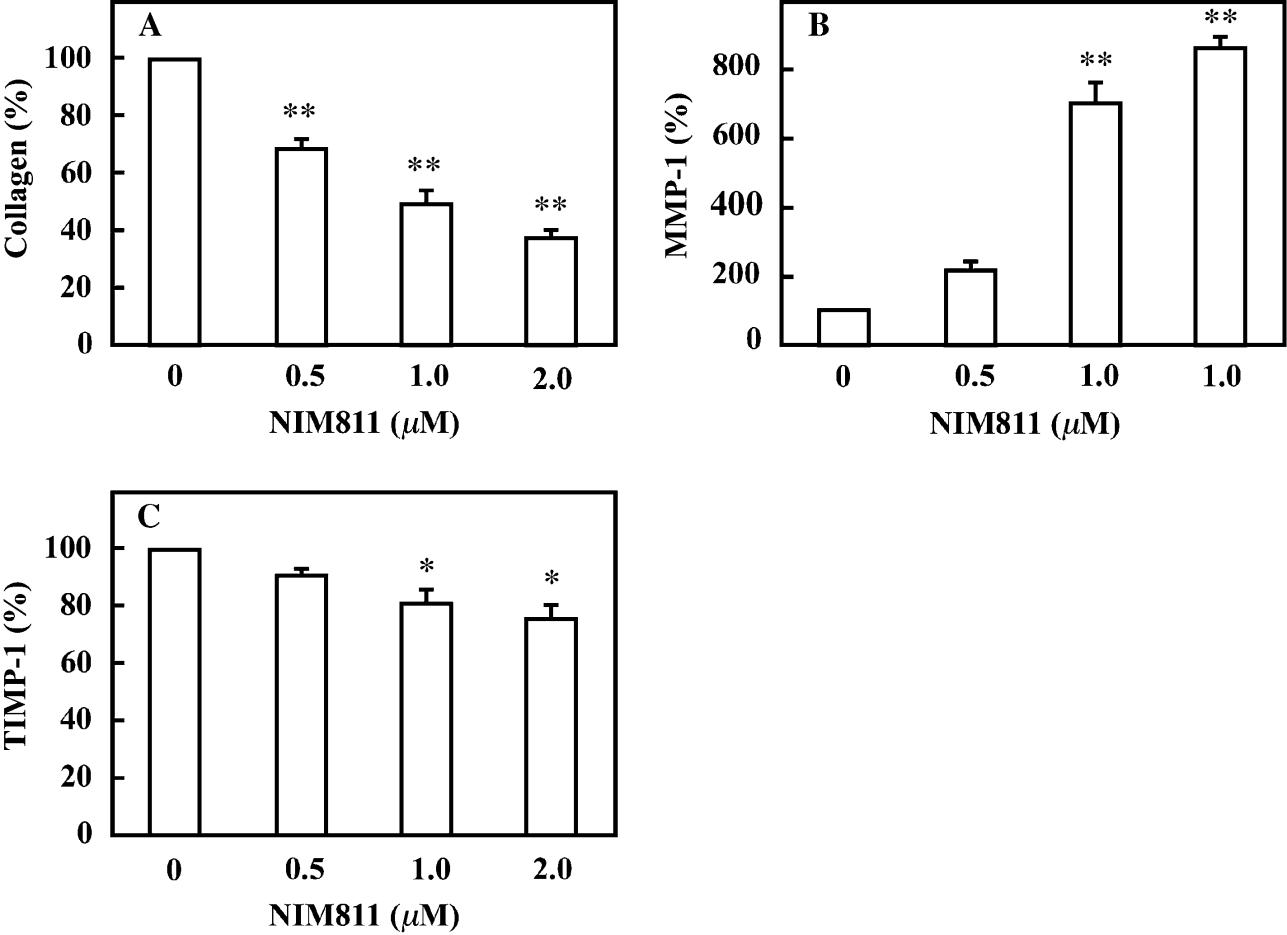
Real-time reverse transcriptase-polymerase chain reaction (RT-PCR) analysis of type I collagen, matrix metalloproteinase 1 (MMP-1) and tissue inhibitor of metalloproteinase-1 (TIMP-1) mRNA expression. Total RNA was extracted from hepatic stellate cells (HSCs) treated with NIM811 (0.5, 1 or 2 mM) or mock treated. Samples were analysed by real-time RT-PCR and all PCR reactions were normalized for glyceraldehyde-3-phosphate dehydrogenase (GAPDH) expression. NIM811 reduced the expression of type I collagen (A) and TIMP-1 (C) but it enhanced the expression of MMP-1 (B). The ratio of expression in the absence of NIM811 was used as a control (100%). The data represent mean±SEM from three independent experiments. ^*^and ^**^Statistically significant differences (*P*<0.05 and *P*<0.01 respectively) compared with HSCs in the absence of NIM811.

We used RT-PCR to evaluate the effects of NIM811 or cyclosporine on the mRNA levels of type I collagen, MMP-1 and TIMP-1. The expression of type I collagen was reduced by roughly 30% in the presence of 0.5 mM NIM811 (Fig. 3A). In contrast, 0.5 mM NIM811 increased the expression of MMP-1 nearly two-fold (Fig. 3B) but did not affect that of TIMP-1 (Fig. 3C). These results indicated that the effects of NIM811 on gene expression were similar to its effect on protein production.

### Effect of NIM811 on cell proliferation and apoptosis

**Fig. 4:**
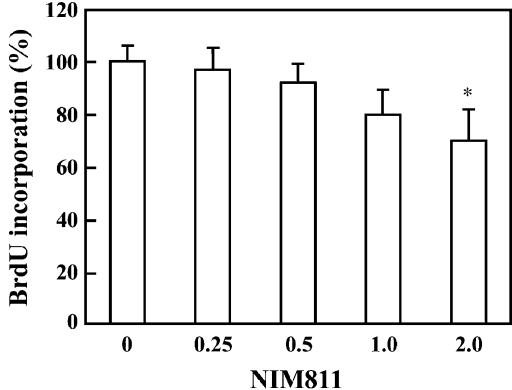
Effects of NIM811 on BrdU incorporation into hepatic stellate cells (HSCs). NIM811 (2.0 mM) decreased BrdU incorporation in cells by nearly 30% compared with untreated cells, although treatment with a lower concentration did not. The data represent mean±SEM from five independent experiments. ^*^Statistically significant difference (*P*<0.05) compared with HSCs in the absence of NIM811.

Bromodeoxyuridine incorporation was measured to investigate the effect of NIM811 on cell proliferation. Quantitative analysis showed that 2.0 mM NIM811 treatment decreased new DNA synthesis by nearly 30%, although treatment with lower concentrations had a reduced effect (Fig. 4). Next, we evaluated the effects of NIM811 on apoptosis; even in the presence of 2 mM NIM811, little apoptosis was observed (data not shown).

### Effects of NIM811 on MAPKs signalling pathways

**Fig. 5:**
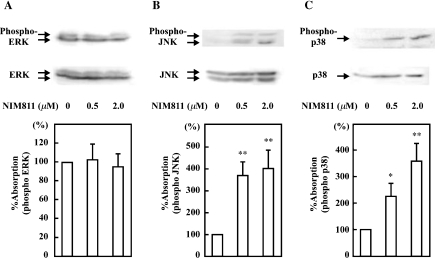
Western blot analysis of phosphorylated and nonphosphorylated extracellular signal-regulated kinases 1 and 2 (ERK1/2) (A), JNK (B) and p38 (C). NIM811 enhanced the phosphorylation of JNK and p38, but not ERK1/2. Each figure is representative of three independent experiments. Band intensities were measured to determine the ratios of phosphorylated to nonphosphorylated ERK1/2, JNK and p38. The ratio of phosphorylation in the absence of NIM811 was used as a control (100%). The values shown are the mean±SEM of triplicate determination. ^*^and ^**^Statistically significant differences (*P*<0.05 and <0.01 respectively) compared with hepatic stellate cells (HSCs) in the absence of NIM811.

Because cell growth and the expression of genes involved in the process of cell growth are widely regulated through MAPK signal cascades, we assessed the effects of NIM811 on MAPK activity, including ERK1/2, JNK and p38. Treatment with NIM811 significantly enhanced the phosphorylation of JNK and p38 MAPK in a concentration-dependent manner, but did not enhance the phosphorylation of ERK1/2 (Fig. 5A–C). NIM811 at a concentration of 0.5 mM enhanced the phosphorylation of JNK and p38 MAPK by nearly 3.6- and 2.3-fold respectively (Fig. 5B and C).

### Effects of NIM811 on tumour growth factor-β signalling pathways

**Fig. 6:**
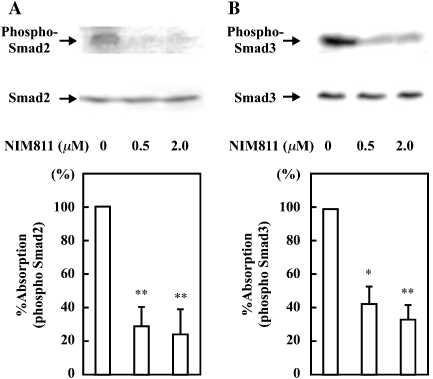
Western blot analysis of phosphorylated and nonphosphorylated Smad2 and Smad3. NIM811 strongly suppressed the phosphorylation of Smad2 (A) and Smad3 (B). Each figure is representative of three independent experiments. Band intensities were measured to determine the ratios of phosphorylated to nonphosphorylated Smad2 and Smad3. The ratio of phosphorylation in the absence of NIM811 was used as a control (100%). The values shown are the mean±SEM of triplicate determination. ^*^and ^**^Statistically significant differences (*P*<0.05 and <0.01 respectively) compared with hepatic stellate cells (HSCs) in the absence of NIM811.

**Fig. 7:**
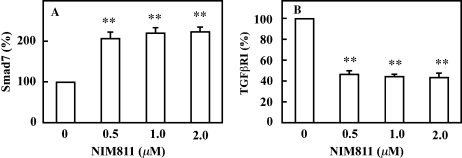
Real-time reverse transciptase-polymerase chain reaction (RT-PCR) analysis of Smad7 and tumour growth factor-β (TGF-β)-RI mRNA expression. Total RNA was extracted from hepatic stellate cells (HSCs) treated with NIM811 (0.5, 1, or 2 mM) or mock treated. Samples were analysed by real-time RT-PCR, and all PCR reactions were normalized for glyceraldehyde-3-phosphate dehydrogenase (GAPDH) expression. NIM811 enhanced the expression of Smad7 (A), and it reduced the expression of TGF-β-RI (B). The ratio of expression in the absence of NIM811 was used as a control (100%). The data represent mean±SEM from three independent experiments. ^**^Statistically significant differences (*P*<0.01) compared with HSCs in the absence of NIM811.

Because TGF-β signal cascades through Smad2 and Smad3 strongly regulate the expression of the type I collagen gene (27), we evaluated the effects of NIM811 on the phosphorylation of Smad2 and Smad3. Treatment with NIM811 significantly suppressed the phosphorylation of Smad2 and Smad3 in a concentration-dependent manner; 0.5 mM NIM811 suppressed the phosphorylation of Smad2 and Smad3 by nearly 70 and 60% respectively (Fig. 6A and B). Next, we evaluated the expression of Smad7, which negatively regulates TGF-β signalling pathways by inhibition of TGF-β-RI phosphorylation (28). 0.5 mM NIM811 enhanced the expression of Smad7 nearly two-fold (Fig. 7A) and it suppressed that of TGF-β-RI by nearly 50% (Fig. 7B).

## Discussion

A recent study by Berenguer *et al.* (3) demonstrated that HCV-infected patients receiving immunosuppressive therapy with cyclosporine were less likely to develop graft cirrhosis than those treated with tacrolimus. We reported previously that cyclosporine at the clinically relevant concentration of 0.125 mM (150 ng/mL) significantly reduced collagen production in HSCs, while tacrolimus at the clinical concentration of 12.5 nM (10 ng/mL) did not (9). In this study, we demonstrated that NIM811 (0.125 mM), as well as cyclosporine, produced a concentration-dependent suppression of collagen accumulation (Fig. 1). We also found that this suppression was regulated at least as far upstream as the transcriptional level because treatment with NIM811 suppressed collagen gene expression. Collagen accumulation, in addition to being determined by the rate of collagen production, is also regulated by collagenase activity, specifically, by the balance between MMP-1 and TIMP-1. We found that treatment of the cells with NIM811 increased collagenase activity, accompanied by stimulation of the transcription and synthesis of MMP-1 and weak suppression of the expression of TIMP-1 (Figs 2 and 3).

Previous work has demonstrated that, in addition to stimulating collagen production, activated HSCs inhibit the degradation of interstitial collagens by collagenases such as MMP-1, indicating that matrix degradation is inhibited during the progression of fibrosis (29–31). TIMP-1 has been reported to regulate cell growth and apoptosis independent of the inhibition of matrix degradation (32). We demonstrated that NIM811 suppressed the growth of HSCs in a concentration-dependent manner without apoptosis (Fig. 4). Taken together, these results indicate that NIM811 has therapeutic potential for liver fibrosis through suppression of collagen production and enhancement of collagenase activity.

To explore the mechanism by which NIM811 suppresses collagen production and cell proliferation, and enhances collagenase activity, we examined the effects of NIM811 on intra-cellular signalling cascades, such as MAPK cascades, which play important roles in collagen production and cell proliferation in HSCs (33). It is intriguing that NIM811 enhanced the activation of JNK and p38 but not ERK1/2 (Fig. 5). In contrast, cyclosporine suppressed the activation of JNK and p38, as we reported previously (9). It was shown that cyclosporine exerts its immunosuppressive effects through both the calcineurin-dependent nuclear factor of activated T cells (NFAT) pathway and the calcineurin-independent activation pathway for JNK and p38 (34). NIM811, an analogue of cyclosporine, does not activate the NFAT pathway because it cannot bind to cyclophilin A (13). The different effects of NIM811 and cyclosporine on JNK and p38 might be attributable to the absence of an effect of NIM811 on the NFAT pathway.

In addition to MAPKs, TGF-β signalling cascades strongly stimulate collagen production by HSCs (28). TGF-β binds to TGF-β-RII on the cell membrane, and then TGF-β-RII phosphorylates TGF-β-RI at the serine and threonine residues located in its glycine-/serine-rich domain (35). The phosphorylated TGF-β-RI phosphorylates Smad2 and Smad3 at a C-terminal SSXS motif and these form a complex with their common partner Smad4. These Smad proteins translocate to the nucleus and activate the transcription of target genes such as collagen (35). In this study, we demonstrated that Smad2 and Smad3 were constitutively phosphorylated in activated HSCs, as reported previously (36), and that NIM811 suppressed the phosphorylation of Smad2 and Smad3 (Fig. 6). These results suggest that NIM811 may inhibit the kinase activity of TGF-β-RII and/or TGF-β-RI. Several molecules such as Smad7 (28, 37), immunophilin FK506-binding protein (FKBP) 12 (38) and Smad anchor for receptor activation (SARA) (39) are associated with TGF-β-R and regulate TGF-β signalling. We found that NIM811 enhanced the expression of Smad7, and suppressed that of TGF-β-RI, indicating that NIM811 inhibits the TGF-β signalling pathways, at least partially through blockade at the receptor level. We also found that cyclosporine had similar effects on Smad2, Smad3, Smad7 and TGF-β-RI (unpublished data). As mentioned above, NIM811 had effects opposite to those of cyclosporine on JNK and p38, although both showed similar effects on collagen production and cell proliferation, suggesting that NIM811 and cyclosporine exhibit antifibrogenic effects mainly by blockade of TGF-β signalling pathways.

Because NIM811 lacks the ability to bind to cyclophilin A (13), NIM811 exerts its pharmacological effects by binding to other cyclophilins, such as cyclophilin B or D. Cyclophilins are a family of PPIases that catalyse the cis-trans interconversion of peptide-bound amino-terminal proline residues, facilitating changes in protein conformation (40). There are more than 10 subtypes of cyclophilin and they are involved in numerous cellular processes, including transcriptional regulation, immune response, protein secretion and mitochondrial function (40, 41). Watashi *et al.* (42) recently reported that NIM811 suppressed the replication of an HCV replicon *in vitro*, whereas tacrolimus did not show this effect. Notably, NIM811 exerts its antiviral effects via binding cyclophilin B, which is a functional regulator of HCV RNA polymerase (43). NIM811 has also been reported to have cytoprotective properties depending on interference with the interaction with cyclophilin D, which regulates the mitochondrial permeability transition (13). Kon *et al.* (44) reported that NIM811 prevented acetaminophen-induced necrosis and apoptosis of cultured mouse hepatocytes. In order to explain the detailed working mechanism of NIM811, cyclophilins interacting with NIM811 are important factors; however, we have not determined which cyclophilin is utilized by NIM811 to exert its anti-fibrogenic and anti-proliferating activity. We are now in the process of identifying the target cyclophilin.

In conclusion, we demonstrated that NIM811, as well as cyclosporine, had anti-fibrogenic effects. NIM811 has no immunosuppressive activity and, in consideration of the toxicity data, seems more favourable for clinical use because of the absence of significant changes in kidney-specific parameters following 10 days of 50 mg/kg of oral NIM811, whereas the same dose regimen of cyclosporine produced signs of renal dysfunction (45). NIM811 would be a plausible candidate for prevention of the progression of HCV-related graft-cirrhosis after liver transplantation because of its anti-viral and anti-fibrogenic effects *in vitro*. Further studies *in vivo* will be required to determine whether NIM811 is effective for the treatment of hepatic fibrosis.
